# NAT2 gene polymorphisms in three indigenous groups in the Colombian Caribbean Coast region

**Published:** 2014-12-30

**Authors:** Isis Arias, Nelly Lecompte, Lila Visbal, Iliana Curiel, Enio Hernández, Pilar Garavito, Carlos Silvera-Redondo

**Affiliations:** 1 Grupo de Investigación en Genética y Medicina Molecular, Departamento de Medicina, Universidad del Norte, Barranquilla Colombia.; 2 CINPE-Centro de Investigación en Neonatología y Pediatría, Departamento de Medicina, Universidad del Norte, Barranquilla Colombia.; 3 Grupo de Investigación en Biomedicina Molecular, Facultad de Medicina, Universidad Cooperativa de Colombia, Santa Marta, Colombia.

**Keywords:** NAT2, single nucleotide polymorphism, genotyping, acetylation, Isoniazid, Chimila, Wiwa, Wayuu, Indigenous groups

## Abstract

**Objective:**

To study the *NAT2* gene polymorphisms 481T, 590A and 857A in the Chimila, Wiwa and Wayuu indigenous groups of the Colombian Caribbean to determine the frequencies of the alleles NAT2*4, NAT2*5, NAT2*6, and NAT2*7 and to determine the types of acetylators present in these populations.

**Methods:**

A total of 202 subjects were studied: 47 Chimila, 55 Wiwa, and 100 Wayuu. The polymorphisms were identified using a real-time PCR method for allelic discrimination designed using Taqman of Applied Biosystems.

**Results:**

The following alleles were found at the highest frequency in the following groups: the NAT2*4 allele (*wild type*) in the Wayuu group (55.3%), the NAT2*5 allele in the Wiwa group (34.5%), and the NAT2*7 allele in the Chimila group (24.2%). A higher frequency of the rapid acetylator status was found in the Wayuu group (31.3%) and Chimila group (29.5%) compared with the Wiwa group (12.7%). The intermediate acetylator status distribution was very similar in all three groups, and the frequency of the slow acetylator status was higher in the Wiwa group (32.7%) compared with the Chimila and Wayuu groups (20.5% and 21.2%, respectively).

**Conclusion:**

The results demonstrated the allelic distribution and pharmacogenetic differences of the three groups studied and revealed the most frequent acetylator status and phenotype. Because of the high prevalence of slow acetylators, a greater incidence of tuberculosis (TB) drug-induced hepatotoxicity is predicted in these populations, with a higher frequency in the Wiwa group.

## Abstract (es)

**Objetivo:**

Estudiar los polimorfismos tipo SNP (*del inglés-* *single nucleotide polymorphism*) 481T, 590A y 857A del gen *NAT2*, en los grupos indígenas Chimila, Wiwa y Wayúu del Caribe Colombiano para determinar las frecuencias de los alelos NAT2*4, NAT2*5, NAT2*6 y NAT2*7 y caracterizar el tipo de acetiladores presentes en estas poblaciones.

**Métodos:**

Se estudiaron 202 individuos en total, 47 Chimila, 55 Wiwa y 100 Wayúu. Los polimorfismos se determinaron mediante la técnica de PCR en tiempo real por el método de discriminación alélica Taqman de *Applied Biosystems.*

**Resultados:**

El alelo NAT2*4 (*wild type*) mostró una mayor frecuencia en el grupo Wayúu (55.3%), el alelo NAT2*5 en el grupo Wiwa (34.5%) y el alelo NAT2*7 en el grupo Chimila (24.2%). Se encontró una mayor frecuencia del estado acetilador rápido en el grupo Wayúu (31.3%) y en el grupo Chimila (29.5%) al compararse con el grupo Wiwa (12.7%). La distribución del estado acetilador intermedio es muy similar en los tres grupos, y para el estado acetilador lento observamos que en el grupo Wiwa la frecuencia es mayor (32.7%) con respecto a Chimila y Wayúu con 20.5% y 21.2% respectivamente.

**Conclusiones:**

Los resultados permitieron conocer la distribución alélica y el componente farmacogenético de los tres grupos estudiados; igualmente, deducir el estado acetilador y/o fenotipo más frecuente. Debido a la alta prevalencia de acetiladores lentos, se podría predecir un aumento de la incidencia de hepatotóxicidad inducida por medicamentos antituberculosos como la Isoniacida indicados en estas poblaciones y en mayor frecuencia en el grupo Wiwa.

## Introduction

The *NAT2* gene codes for the enzyme arylamine N-acetyltransferase 2 (NAT2), which is involved in phase II of the detoxification and metabolization of xenobiotics and components that contain aromatic amines by N- or O-acetylation. Furthermore, it also metabolizes antibiotics, such as isoniazid, which is used to treat tuberculosis (TB) 1 ^-^ 3. 

The *NAT2* gene locus has been identified as 8p22, and currently, 59 allele variants have been described in different human populations. These variants have between one and four nucleotide substitutions in positions 191, 282, 341, 434, 481, 590, 803, 845, and 857; with the exception of positions 282 and 481, the remaining seven variations result in an amino acid change 4 ^-^ 7.

There are four main alleles of the *NAT2 *gene. NAT2*4, is known as the *wild-type* allele, and NAT2*5, NAT2*6, and NAT2*7 are mutant alleles with the single nucleotide polymorphisms (SNPs) 481C>T, 590G>A, and 857G>A, respectively. The nucleotide change at position 481 does not result in a change in the coded amino acid; in position 590, there is a change from arginine (Arg) to glutamine (Gln); and in position 857, there is a change from glycine (Gly) to glutamic acid (Glu) 8.

Depending on the enzymatic activity, 3 types of acetylators have been defined. *Rapid acetylators* have two normal alleles with the genotype NAT2*4/*4; *intermediate acetylators *have a normal and mutant allele with the possible genotypes NAT2*4/*5, NAT2*4/*6, and NAT2*4/*7; and *slow acetylators* have two mutant alleles with the possible genotypes NAT2*5/*5, NAT2*5/*6, NAT2*5/*7, NAT2*6/*6, NAT2*6/*7, and NAT2*7/*7 (Table 1) 4 ^,^ 9. 

**Table 1.**
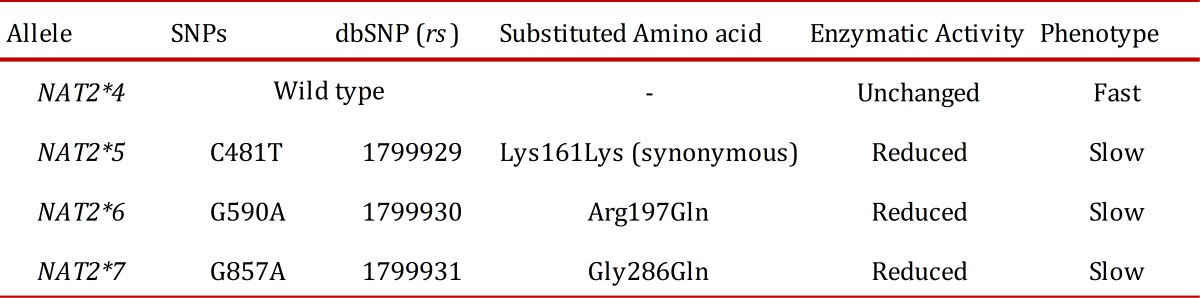
N-acetyltransferase 2 (NAT2) the single nucleotide polymorphisms (SNPs)

The frequency of the acetylator phenotype differs depending on the ethnic group; the slow acetylator phenotype is found in 40-60% of Caucasians, 60% of African-Americans, 10-20% of Asian people, 5% of Inuit people, and 90% of Mediterranean populations 7 ^,^ 10.

Studies on indigenous people from Argentina and Paraguay have reported a >40% frequency for the NAT2*4 allele (42.9-80.0%). Similarly, the allele NAT2*5 was found at a frequency between 31.2% and 50.0% in the Mapuche and Tehuelche groups (Patagonia, Argentina) and the Lengua and Ayoreo groups (Paraguay). The NAT2*6 allele was found in 3% of Wichi people and in more than 12% of the Jujuy, Mapuche, and Tehuelche populations, whereas the NAT2*7 allele had high variability, from 4.5% to 42.9%, among the different populations 10. In their study, Jorge-Nebert *et al*., reported that the Ngawbe and Embera populations had frequencies of 2.4% and 9.9% for the allele NAT2*5, 0% and 3.7% for the allele NAT2*6, and 23.3% and 22.8% for the allele NAT2*7, respectively 11. In a recent study on the native people of Brazil, it was reported that the Tupinamba group had a 43.3% frequency of the alleles NAT2*5 and NAT2*6 and a 10% frequency of the allele NAT2*7 12.

Given that SNPs at positions 481, 590 and 857 are the cause of 95% of the low enzymatic activity alleles 1 ^,^ 4 ^,^ 9 and their identification can be used to determine the acetylating status of any individual, the aim of this study was to analyze these polymorphisms in the indigenous Chimila, Wiwa, and Wayuu groups, which are representative of the Colombian Caribbean Coastal region, to determine the allelic and genotypic frequencies and thus the acetylating types present among these populations.

## Materials and Methods

### Population

The present study included 202 individuals from three indigenous groups that inhabit the Colombian Caribbean Region. The samples obtained took into account the phenotypic traits characteristic of these indigenous populations (thin, straight black hair, dark eyes, red tinted skin, and average stature), their geographic location, the preservation of their traditional activities and rites according to their socio-cultural behavior and originating during the pre-Hispanic time, and the low consanguinity grade. The larger groups of the indigenous group *Ette ennaka* or Chimila (own people), also known in ethnographic literature as Simiza, Chimíle, Simza, or Shimizya (Preuss, 1926; Ortiz, 1965 and Loukotka, 1968, cited by^13^), are located in the central prairies of the Department of Magdalena, which is in the *Naara Kajmanta* settlement in Puerto Mosquito bordering the Sierra Nevada de Santa Marta and located in Sabanas de San Angel county protected by the Issa Oristunna reservation (Land of New Hope). These groups speak the *ette taara* (tongue of the people) language, which belongs to the linguistic family Chibcha. Their population is estimated to be 910 individuals 13 ^-^ 16. The indigenous group Wiwa, also known as Arsario, Guamaca, Malayo, Sanjá, or Dumana, currently resides in the town called El Encanto (Gotsezhi) in Guachaca, which borders the Sierra Nevada de Santa Marta, Department of Magdalena. Their mother tongue is *damana*, which belongs to the linguistic family Chibcha, and their population is estimated to be 13,627 individuals 15 ^-^ 17. The indigenous group Wayuu (lord, powerful man), also known as Wayu, Uáira, or Waiu, are characterized by a broad nose, dark eyes, long straight hair, average stature, and stout physique. They inhabit the high and intermediate part of the Guajira peninsula in the Caribbean Sea, Manaure County, Department of Guajira. Their denominate language is *wayuunaiki *from the linguistic family Arawak. Their Colombian population is estimated to be 149,827 individuals 15 ^,^ 18. This study was evaluated and approved by the Comite de Bioetica en Investigacion de la Universidad Cooperativa de Colombia, in Santa Marta (resolution 024 from 2010). All of the participating individuals gave their informed consent prior to providing samples.

### Genotyping

A sample of peripheral blood was collected from each individual and placed in a test tube containing EDTA. Genomic DNA was isolated from lymphocytes using an UltraCleanTM Blood kit (Mo-Bio). The three SNPs, C481T (rs1799929), G590A (rs1799930), and G857A (rs1799931), were analyzed by qRT-PCR using TaqMan allelic discrimination and the assays C_1204092_20, C_1204091_10, and C_572770_20, respectively, in an ABI PRISM 7500 Real-Time PCR System (Applied Biosystems). These assays detect the four main alleles: NAT2*4, NAT2*5, NAT2*6, and NAT2*7 19. The experimental reactions were conducted in a final volume of 25 μL and included the following components: 20 ng of genomic DNA; 12.5 μL of TaqMan Universal PCR Master Mix with AmpliTaq Gold DNA polymerase, AmpErase^®^ Uracil N-glicosilase (UNG), deoxynucleotide mix (dNTPs) with dUTP, Passive reference (ROX), and Buffer; and 1.25 μL of 20X Drug Metabolism Genotyping Assay Mix (specific for each polymorphism) containing 18 μM of each primer and 4 μM of each probe (VIC/FAM). All experiments were performed following the same amplification and detection protocol, which consisted of 50º C for 2 min, 95º C for 5 min, 50 cycles of 92º C for 10 s and 60º C for 90 s 19. The polymorphisms were determined according to the amplification curves recognized for each probe (VIC/FAM).

### Statistical analysis

The statistical analyses of the allelic and genotypic frequencies were conducted using a Chi-squared test with the SPSS^®^ (*Statistical Package for the Social Science*) program for Windows v.19.0. The frequency comparison for a single mutation, allelic and genotypic, between the studied populations was conducted using the statistics program Arlequin^® ^ v.3.5. The expected genotype frequency was calculated using the Hardy-Weinberg equation. Finally, a comparison between the allelic and genotypic frequencies found and those previously reported was conducted.

## Results

The studied population consisted of 47 indigenous Chimila (51% males and 49% females), 55 indigenous Wiwa (53% males and 47% females), and 100 indigenous Wayuu (11% males and 89% females). According to the analysis of each analyzed SNP in the total study population, the allele 481T had the highest frequency (26.5%) followed by 857A (18.1%), and the least frequent allele was 590A (6.9%). The allelic and genotypic frequencies for the *NAT2* SNPs found in the total studied population and in each indigenous group are listed (Table 2).

**Table 2.**
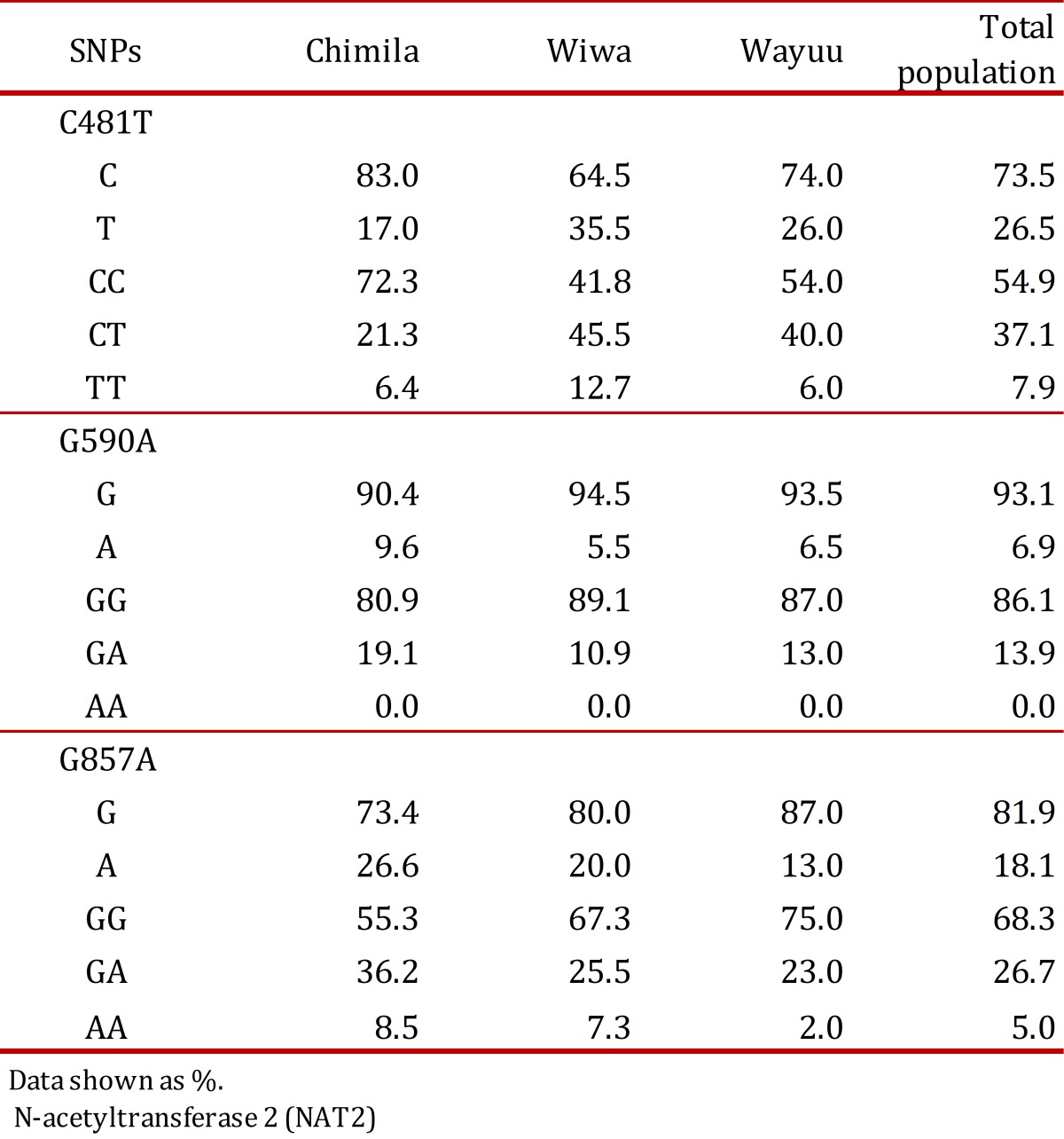
N-acetyltransferase 2 (NAT2) the single nucleotide polymorphisms (SNPs) frequencies in the study groups.

The allele NAT2*4 (*wild type*) was found in 50.3% of the total population and more frequently in the Wayuu group (55.3%). Among the mutant alleles, the most frequently found was NAT2*5 (26.3%), and the allele NAT2*6 was found in only 6.0% of the population.

Regarding each studied group, SNP 481T was the most frequent in the Wiwa group (34.5%); SNP 590A was found more frequently in the Chimila group (9.6%); and SNP 857A was found at a high frequency in both the Chimila and Wiwa groups (26.6% and 20.0%, respectively). Regarding the alleles, NAT2*5 was found at a high frequency in the Wiwa group (34.5%), and the allele NAT2*7 was found at a higher frequency in the Chimila group (24.2%), as shown in Table 3.

**Table 3.**
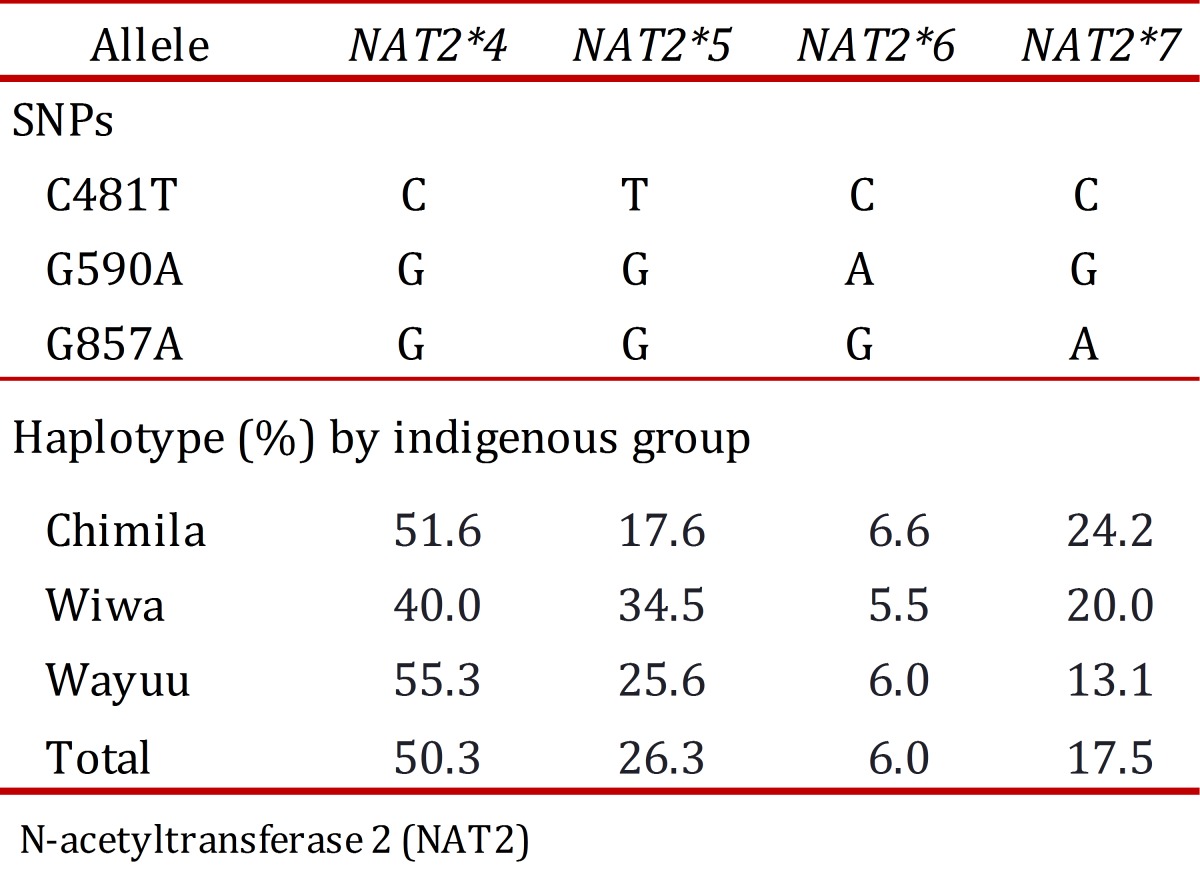
Allele frequency in the Chimila, Wiwa and Wayuu indigenous groups.

The distribution of the *NAT2* alleles was found to be at equilibrium in the three studied groups according to the Hardy-Weinberg equation (*p*> 0.05).

Based on the identified allelic combinations, eight genotypes were present in the analyzed population with the exception of NAT2*6/*6 and NAT2*6/*7. The genotype NAT2*4/*4, associated with a rapid acetylator status, was the most frequent (25.2%) in the three studied groups compared with the other genotypes.

The genotype NAT2*4/5, an intermediate acetylator, was found at a higher frequency in the Wiwa group (32.7%) compared with the Chimila (9.1%) and Wayuu groups (27.3%). All of the genotypes specifying a slow acetylator status had similar distribution patterns (2-11%), with the most frequent being NAT2*5/*5 (10.9%) in the Wiwa group, closely followed by the Wayuu group (10.1%).

The frequency of a rapid acetylator status was higher in the Wayuu (31.3%) and Chimila (29.5%) groups compared with the Wiwa group (12.7%). The distribution of the intermediate acetylator status was very similar among the three studied groups. We found the highest frequency of the slow acetylator status in the Wiwa group (32.7%) compared with the Chimila (20.5%) and Wayuu (21.2%) groups (Table 4).

**Table 4.**
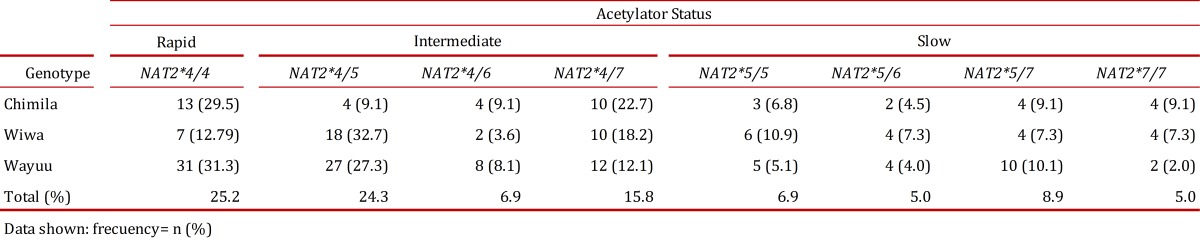
Acetylator status in the Chimila, Wiwa and Wayuu indigenous groups.

## Discusion

The consecutive study of the *NAT2* gene in a large number of populations revealed that this gene has wide allelic diversity and inter-ethnic variation, which is reflected in the different types of acetylators present in the analyzed population.

The frequency of the acetylator phenotypes differs according to the ethnic group. For example, the frequency of a slow acetylator phenotype is 40-60% in Caucasians, 10-20% in African-Americans, 5% in Asians, 5% in Inuit, and 90% in certain Mediterranean populations. The frequency of intermediate acetylators is 35.7% in Caucasians, 45.9% in Japanese people, and 46.6% in the Chinese population 20 ^-^ 22. The frequency of rapid acetylators is 25% in Caucasians and 70% in Japanese people 23 ^,^ 24. Caucasian and African populations have a high NAT2*5 allele frequency (28%) and a low NAT2*7 allele frequency (7%), whereas Asian populations have a low NAT2*5 allele frequency 1 ^,^ 5 ^,^ 8 ^,^ 10.

Studies performed on South American indigenous groups in Argentina and Paraguay have reported a >40% frequency for the NAT2*4 allele (42.9-80%); the NAT2*5 allele was also found at high frequencies of 31.2% and 50% in the Mapuche and Tehuelche groups (Patagonia, Argentina) and in the Lengua and Ayoreo groups (Paraguay), respectively. The NAT2*6 allele was found at a frequency of 3% in the Wichi group and in more than 12% of the Jujuy, Mapuche, and Tehuelche populations, and the NAT2*7 allele has demonstrated high variability between the different populations (4.5%-42.9%) 10. 

Conversely, in 2002, Jorge-Nebert *et al*., reported frequencies of 2.4% and 9.9% for the NAT2*5 allele, 0% and 3.7% for the NAT2*6 allele, and 23.3% and 22.8% for the NAT2*7 allele in the Ngawbe and Embera populations, respectively 11. In a recent study performed on native people from Brazil, it was found that the Tupinamba group had a 43.3% frequency for the NAT2*5 and NAT2*6 alleles and a 10% frequency for the NAT2*7 alleles 12.

The results from the analysis of three different populations in this study revealed that despite sharing a close geographical location and similar languages (Chibcha, Wiwas and Chimilas), there were important differences in the distribution patterns of the alleles and phenotypes. These types of differences, however, have also been observed in the analysis of other indigenous groups in South and Central America (such as Embera, Ngawbe, and Tupinamba) 11.

In the three studied groups, the NAT2*4 allele had a higher frequency (Chimila: 51.6%, Wiwa: 40.0%, and Wayuu: 55.3%), which does not represent a difference between the groups. The NAT2*6 allele had a similar distribution pattern in the three groups (Chimila: 6.6%, Wiwa: 5.5%, and Wayuu: 6.0%). Inter-group differences were found for the NAT2*5 allele (Chimila: 17.6%, Wiwa: 34.5%, and Wayuu: 25.7%). Similarly, the NAT2*7 allele was found at a lower frequency in the Wayuu (13.0%) compared with the Chimila (24.2%) and the Wiwa (20.0%). The organization of the alleles in the genotypes allowed us to decipher the acetylator status in the different groups. Based on our findings, the Wiwa had a low percentage of rapid acetylators (12.7%) compared with the Chimila (29.5%) and the Wayuu (31.3%) because we detected a high frequency of slow acetylator individuals among the general population.

When comparing the obtained results with other indigenous groups (Table 5), the NAT2*4 allele in the studied populations, as in other populations from Panama (Ngawbe and Embera) and Brazil (Amerindians), was found at a higher frequency compared with the mutant alleles NAT2*5, NAT2*6, and NAT2*7. The NAT2*5 allele was found at a higher frequency in the Wiwa group (34.5%) compared with the other two studied groups, and this frequency was considered to be even higher compared with the Ngawbe and Embera groups (2.4% and 9.2%, respectively) 11. The NAT2*6 allele was found at a high frequency in all three studied groups compared with the Embera group (3.7%), and it was absent in the Ngawbe group. Moreover, the NAT2*7 allele was found in the Chimila (24.7%) and the Wiwa (20.0%) at frequencies similar to the native populations of Panama (Ngawbe and Embera) 11 ^,^ 12.

The slow acetylator status was found more frequently (~50%) in the studied groups compared with the reported frequencies for the Ngawbe (7.6%) and Embera groups (14.7%) 11.

This study on *NAT2* polymorphisms in the analyzed populations demonstrates the large genetic variability in these groups, which is independent of language and geographical location and is potentially due to the low mixing rate between the populations, which also contributes to the socio-cultural and economic conditions. The findings from studies are important for pharmacokinetic studies on Colombian Caribbean populations and are especially important for their potential contribution to the treatment of TB, which is highly prevalent in these populations. The drugs used to treat TB are metabolized specifically by this gene. 

**Table 5.**
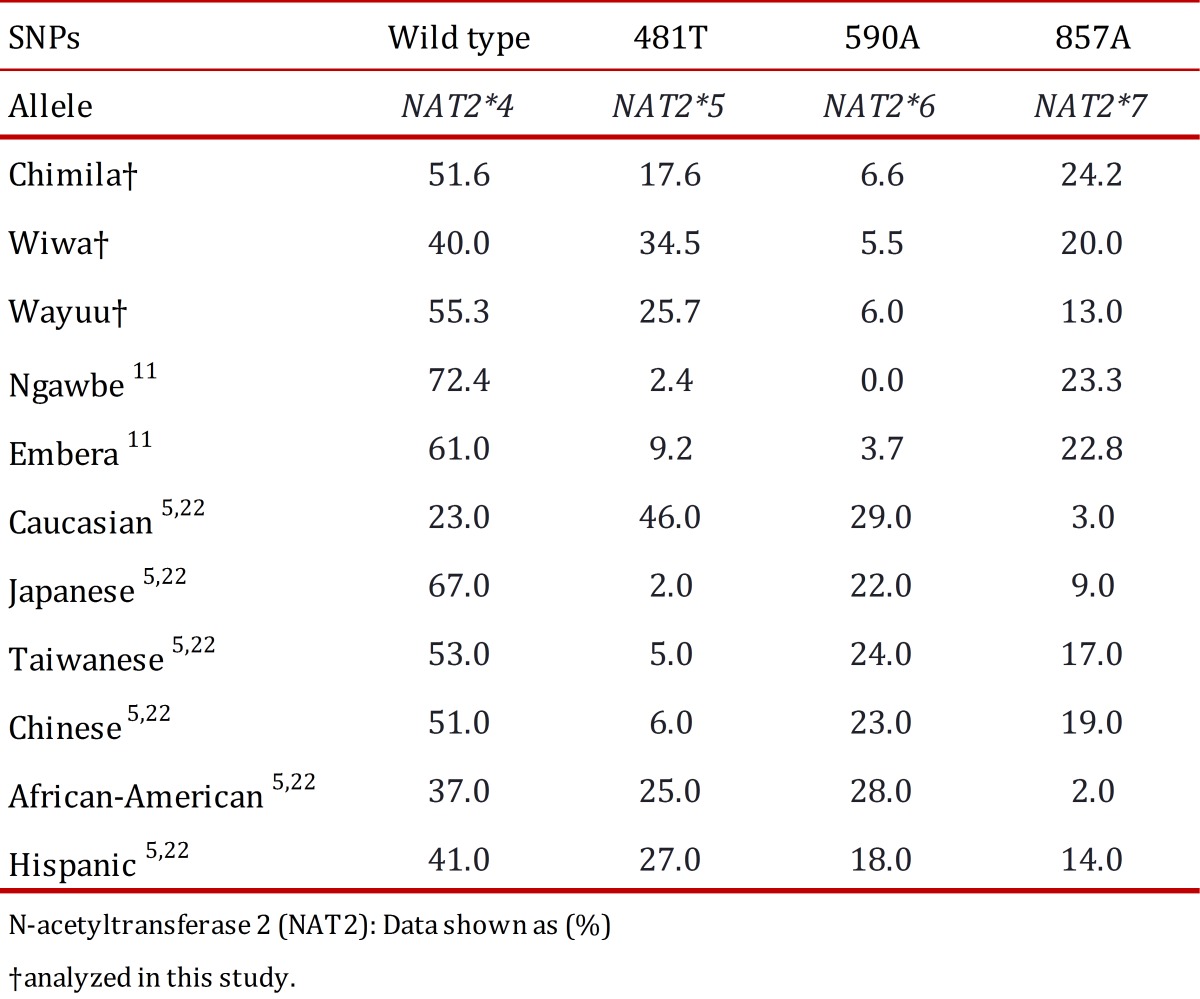
N-acetyltransferase 2 (NAT2) allele frequencies in the Chimila, Wiwa and Wayuu indigenous groups and other populations.

## Conclusion

By analyzing the *NAT2* polymorphisms 481T, 590A, and 857A, we were able to ascertain the allelic distributions and pharmacogenetic differences in the Chimila, Wiwa, and Wayuu groups. Accurately predicting the most frequent acetylator status is useful for selecting the correct therapeutic drugs and their appropriate dose for treating several diseases that are targeted by NAT2-metabolizing drugs. Moreover, it was concluded that the high prevalence of slow acetylators may contribute to the high incidence of TB drug-induced hepatotoxicity in these populations.
